# Seasonal malaria chemoprevention and mutations in *Pfdhfr* and *Pfdhps* genes in children in the health district of Nanoro, Burkina Faso

**DOI:** 10.5281/zenodo.15039792

**Published:** 2025-03-17

**Authors:** Kié Solange Millogo, Adjaratou Zabré, Paul Sondo, Bérenger Kaboré, Amélé Fifi Chantal Kouevi, Eulalie W. Compaoré, Ipéné Mylène Carenne Bayala, Bouda Ismaïla, So-vii Franck Hien, Toussaint Rouamba, Adama Kazienga, Karim Derra, Eli Rouamba, Marc Christian Tahita, Florence Ouédraogo, Hamidou Ilboudo, Sanata Bamba, Halidou Tinto

**Affiliations:** 1 Institut de Recherche en Sciences de la Santé (IRSS)/ Clinical Research Unit of Nanoro (CRUN), Nanoro, Burkina Faso.; 2 Institut Supérieur des Sciences de la Santé (INSSA)/Université Nazi Boni, Bobo Dioulasso, Burkina Faso.

## Abstract

**Introduction:**

Seasonal malaria chemoprevention (SMC) is an effective malaria preventive intervention in sub-Saharan Africa. As with other drug-based interventions, large-scale deployment increases drug pressure, which may result in drug-resistant parasite strains thereby jeopardising the impact of the intervention. Mutations in *Pfdhps* and *Pfdhfr* genes are known to be associated with resistance to sulfadoxine and pyrimethamine, respectively, making the surveillance of molecular markers crucial in settings where SMC is widely applied. This study aimed at assessing the distribution of *Pfdhfr* and *Pfdhps* alleles before and after the 2021 annual campaign of SMC in the health district of Nanoro in Burkina Faso.

**Materials and Methods:**

Randomly selected dried blood spots collected prior (n=100) and after (n=100) the 2021 SMC campaign were used for the detection of mutation in codons 51, 59 and 108 of the *Pfdhfr* gene and in codons 437 and 540 of *Pfdhps* gene using a nested PCR with restriction fragment length polymorphism approach.

**Results:**

The prevalence of *Pfdhfr* and *Pfdhps* mutant alleles were very high before and after SMC, ranging from 88.42% to 97.98%. However, no significant change in the prevalence of *Pfdhfr* and *Pfdhps* mutant alleles was observed in the period before and after SMC campaign (p>0.05). No mutation was observed in *Pfdhps* codon 540. In addition, the prevalence of the *Pfdhfr* triple mutant and *Pfhfr*-*dhps* quadruple mutant was higher in the study area but with no significant variation before and after SMC campaign (p>0.05). .

**Conclusions:**

The prevalence of *Pfdhfr* and *Pfdhps* mutant alleles were higher either in pre or post SMC. However, no significant variation in the prevalence of these alleles was observed following the SMC campaign suggesting that these high mutation frequencies may be the result of continuous use of SMC in Burkina Faso since 2014.

## Introduction

Malaria remains a significant global health problem [1]. In Burkina Faso, it was the first cause of consultation (37.8%), hospitalisation (63.16%) and death (18.2%) in health facilities in 2022 [2]. In the same year, the twelve High Burden to High Impact (HBHI) countries, including Burkina Faso, accounted for 67% of all cases and 73% of malaria related deaths globally [3]. To overcome this health challenge, Burkina Faso has signed up to global initiatives and commitments to combat malaria through the implementation of innovative preventive and curative interventions targeting the most vulnerable populations, particularly children under five and pregnant women. Since 2012, WHO recommends Seasonal Malaria Chemoprevention (SMC) in areas of high seasonal malaria transmission in the Sahel sub-region of Africa to prevent malaria infection in children. Since 2014, SMC is implemented in Burkina Faso and consists of the administration of a full treatment course of amodiaquine + sulfadoxine-pyrimethamine (AQSP) in children aged 3–59 months on a monthly basis during the high transmission season over four months (from June /July to October) [4]. A large study reported that SMC prevented over 88% of uncomplicated malaria cases within 28 days of administration [5]. This high effectiveness is partly attributable to sulfadoxine–pyrimethamine (SP), which remains at a sufficient concentration to inhibit the development of successful blood-stage infections for long periods post treatment [6]. Indeed, a prophylactic period of roughly 42 days against SP sensitive parasites, but shorter for less sensitive parasites was reported [5,6]. SP is a cornerstone of malaria chemoprophylaxis across the African continent [7]. This antimalarial drug is also used for intermittent preventive treatment of malaria in pregnant women (IPTp) and infants (IPTi). However, studies showed that the efficacy of SP for these interventions is influenced by drug resistance levels resulting in SP pressure in parasite populations [7,8]. The massive exposure to SP exerts powerful selection pressure favouring less sensitive strains [9]. Consequently, widespread use of SP for SMC increases drug pressure, which could lead to the emergence and spread of resistant strains within the circulating parasite population, affecting SP efficacy and then the expected impact of the SMC intervention.

Dihydropteroate synthetase (*Pfdhps*) and dihydrofolate reductase (*Pfdhfr*), two enzymes involved in the folate biosynthesis pathway, are synergistically and preferentially targeted by the SP combination [11]. Point mutations in the dihydrofolate reductase gene (*Pfdhfr*) at codons N51, C59, S108, and I164 confer resistance to pyrimethamine while point mutations in the dihydropteroate synthase gene (*Pfdhps*) in codons S436, A437, K540, A581, and A613 are associated with resistance to sulfadoxine in the *P. falciparum* parasite [12]. In Africa, the combined haplotype GE-IRN, comprising mutations in both *Pfdhps* (encoding 437Gly and 540Glu [GE]) and *Pfdhfr* (51Ile, 59Arg, and 108Asn [IRN]), is known to be strongly associated with SP resistance [13,14].

Clinical trials assessing the efficacy of drugs used in prevention strategies especially SP and AQSP are limited in our study area, making the surveillance of molecular markers crucial in settings where SMC and IPTp are widely applied. This study aimed at assessing the distribution of *Pfdhfr* and *Pfdhps* alleles before and after the 2021 SMC annual campaign in the health district of Nanoro in Burkina Faso.

## Materials and Methods

The study was carried out in a rural area of the central part of Burkina Faso in the health district of Nanoro. The area is characterised by a Sudano-Sahelian climate with two seasons: a rainy season occurring from June to October, with proliferation of malaria vectors, and a dry season from November to May, characterised by the harmattan, during which there is an increase of respiratory infections. This climate variability determines the seasonality of malaria transmission which peaks during the rainy season followed by a long low transmission period during the dry season, making the area an ideal place for SMC intervention which has been implemented in the area since 2016 [15]. In the health district of Nanoro, an SMC campaign is implemented yearly from July to October on a monthly basis i.e. 4 rounds/per year. *Plasmodium falciparum* represents the major species (90%), followed by *P. malariae* (3-8%) and *P. ovale* (0.5-2%) [16].

### Sample collection

The dried blood spots (DBS) used in this study were collected in June and November 2021. Samples were collected from children under SMC coverage of two independent studies: SMC-NUT trial (NCT04238845) for samples collected in June and the SMC-RST trial (NCT04816461) for samples collected in November. Details of these two trials are available elsewhere [17,18]. During each of these trials, blood samples were taken from children under SMC coverage for haemoglobin measurement using Hemocue® 801+ (SOC-HE121916, Danayer group, Angelholm, Sweden), for malaria diagnosis by rapid diagnosis tests (RDTs), and microscopy. Malaria slides were stained with 3% Giemsa for 30 min and double read using CX21 microscope (Olympus Corporation, Tokyo, Japan). Drops of blood were spotted onto Whatmann Paper for polymerase chain reaction (PCR) analyses. The dried blood spots were first placed in plastic bags in which silica gel was added to ensure good storage. They were then stored in boxes and kept at room temperature in the laboratory.

### Molecular analysis

*Plasmodium* DNA was extracted from randomly selected DBS before the SMC campaign (n=100) and after the campaign (n=100) using the tween®20-chelex®100 resin method. Three- and two-point mutations in the *Pfdhfr* gene (108, 51 and 59) and the *Pfdhps* gene (437 and 540), respectively, were analysed. PCR and the restriction fragment length polymorphism protocol (PCR-RFLP) were used for the detection of mutations as described elsewhere. The primary and secondary amplification were done by adding a 2μl DNA template to a 23μl reaction mix (here the mix reagents: 14.5μl of DNA water free, 6.5 μl of master mix ready to load (SOLIS BIODYNE, Estonia) and 1 μM of each primer). The products of the secondary PCR containing the polymorphic region were subjected to enzyme digestion for the detection of mutations at the various sites. The following restriction enzymes (New England Biolabs Inc, Ipswich, Massachusetts, USA) were used: for *Pfdhfr*, MluCI, XmnI, and AluI to identify N51I, C59R, and S108N mutations, respectively; for *Pfdhps*, MwoI and FokI to identify A437G and K540E mutations, respectively. *P. falciparum* DNA from laboratory strain 3D7, HB3 and DD2 were included in each PCR reaction and RLFP to serve as positive and negative controls. After digestion, DNA bands were visualised in ethidium bromide-stained 2.5% agarose and alongside a 100 bp molecular weight maker and visualised under a UV transilluminator (Biotec-Fischer GmbH, Reiskirchen, Germany). The results were then classified as wild type, mutant or mixed (when both alleles were present). Cases of mixed infection were classified as mutants.

### Statistical analysis

A descriptive analysis using proportions for qualitative variables was performed. For quantitative variables, means (± standard deviation) were used when data was normally distributed, if not, the median (Q1-Q3) was used. Geometric means and the corresponding 95% confidence interval were used to describe parasite density. Chi-square or Fisher exact tests were used to compare the proportions of different alleles and haplotypes before and after the SMC intervention. The Mann-Whitney U test was used to determine whether the geometric means of the parasite density of the mutant alleles and haplotypes differed from those of the wild type. Data were analysed using R-4.4.2 software and a p-value <0.05 was considered as statistically significant.

## Results

Before the 2021 SMC campaign, the median age of participants was 4.04 years (3.35-4.9) and this was 2.71 years (1.69-3.68) after the SMC campaign. Females represented 56% of the participants before SMC and 49% after the SMC campaign. The mean haemoglobin levels observed were 9.89 ± 1.59 g/dl and 9.16 ± 1.63 g/dl before and after SMC, respectively. The mean temperatures were 36.8 ±0.83 °C and 36.7 ±0.67 °C before and after the SMC campaign. The geometric means of parasite density was 1098.7/μl (95%CI=834.8-1145.9) and 1941/μl (95%CI=1273-2959) before and after SMC, respectively.

### Prevalence of *Pfdhfr* and *Pfdhps* alleles before and after the SMC campaign

Table 1 shows the prevalences of the different alleles in codons 437, 540 of the *Pfdhps* gene and in codons 51, 59 and 108 of *Pfdhfr* gene. Out of the 200 samples analysed, 189 (94.5%) were successfully genotyped for the *Pfdhps* gene including 90 samples before SMC and 99 samples after SMC, giving a genotyping success rate of 90% and 99%, respectively. For *Pfdhfr* gene mutation points, the genotyping was successful in 94% of samples at codon 51 and in 95% at codons 59 and 108 before SMC.

**Table 1:** Period-based comparison of *Pfdhps* (A437G, K540E) and *Pfdhfr* (N51I, S108N, C59R) alleles.

Alleles		Before SMC % (n/N)	After SMC % (n/N)	P-value
A437G				0.32
	Wild	10 (9/90)	6.06 (6/99)	
	Mutant	90 (81 /90)	93.94 (93/99)	
K540E				-
	Wild	100 (90/90)	100 (99/99)	
	Mutant	0(0)	0(0)	
N51I				0.09
	Wild	7.45 (7/94)	2.02 (2/99)	
	Mutant	92.55 (87/94)	97.98 (97/99)	
S108N				0.68
	Wild	2.11 (2/95)	4.04 (4/99)	
	Mutant	97.89 (93/95)	95.96 (95/99)	
C59R				0.17
	Wild	11.58 (11/95)	6.06 (6/99)	
	Mutant	88.42 (84/95)	93.94 (93/99)	

n = number of mutant or wild-type alleles; N = number of samples successfully genotyped. P-values are based on the chi-square or Fisher exact test.

After SMC, the success rate was 99% of samples for all three codons.

High mutation frequencies in *Pfdhps* gene were observed in the study area either before or after the SMC campaign. A437G mutation was observed in 90% (81/90) of samples before the SMC campaign. After the implementation of the SMC campaign, the prevalence of the mutation remained high representing 93.94% (93/99). No mutation 540E was observed.

For the *Pfdhfr* gene, mutations N51I, C59R, and S108N were observed in 92.55% (87/94), 88.42% (84/95) and 97.89% (93/95), respectively, before the SMC campaign. After the campaign, the prevalence of the mutation remained high representing 97.98 % (97/99) for N51I, for C59R and 95.96% (95/99) for S108N, respectively. No significant difference was observed between the prevalences before and after the SMC campaign.

### Prevalence of Pfdhfr and Pfdhfr-dhps haplotypes before and after the SMC campaign

The mutation at *Pfdhfr* N51I, S108N was defined as a double mutant, *Pfdhps* A437G, K540E as double mutants, *Pfdhfr* N51I, C59R, and S108N as triple mutants, *Pfdhfr* N51I, C59R, S108N and *Pfdhps* A437G as quadruple mutants, and the combination of *Pfdhfr* N51I, C59R, S108N plus *Pfdhps* A437G and K540E as quintuple mutants.

The prevalence of double, triple and quadruple mutations were compared before and after SMC campaign (Table 2).

**Table 2:** Comparison of the prevalence of *PfdhfrPfdhps* haplotypes before and after the SMC campaign.

Haplotypes	Before SMC % (n/N)	After SMC % (n/N)	P-value
*Dhfrjlouble*	91.49 (86/94)	93.94 (93/99)	0.51
*Dhfrjriple*	80.65 (75/93)	88.89 (88/99)	0.11
*Dhfr-dhps quadruple*	73.03 (65/89)	83.67 (82/98)	0.08

High frequencies of *Pfdhfr* and *Pfdhfr-dhps* mutant haplotypes were observed in the study area either before or after the SMC campaign. Double, triple and quadruple mutants were observed in 91.49% (86/94), 80.65% (75/93) and 73.03% (65/89) of samples before the SMC campaign, respectively. After the SMC campaign, the prevalence of the mutant haplotypes remained high representing 93.94% (93/99), 88.89% (88/99) and 83.67% (82/98) for *Pfdhfr* double, *Pfdhfr* triple and *Pfdhfr-dhps* quadruple mutants, respectively. No significant difference was observed between the prevalences of these haplotypes before and after SMC campaign.

### Comparison of geometric mean parasite density between *Pfdhfr* and *Pfdhps* mutant and wild type alleles and haplotypes

**Figure 1:**
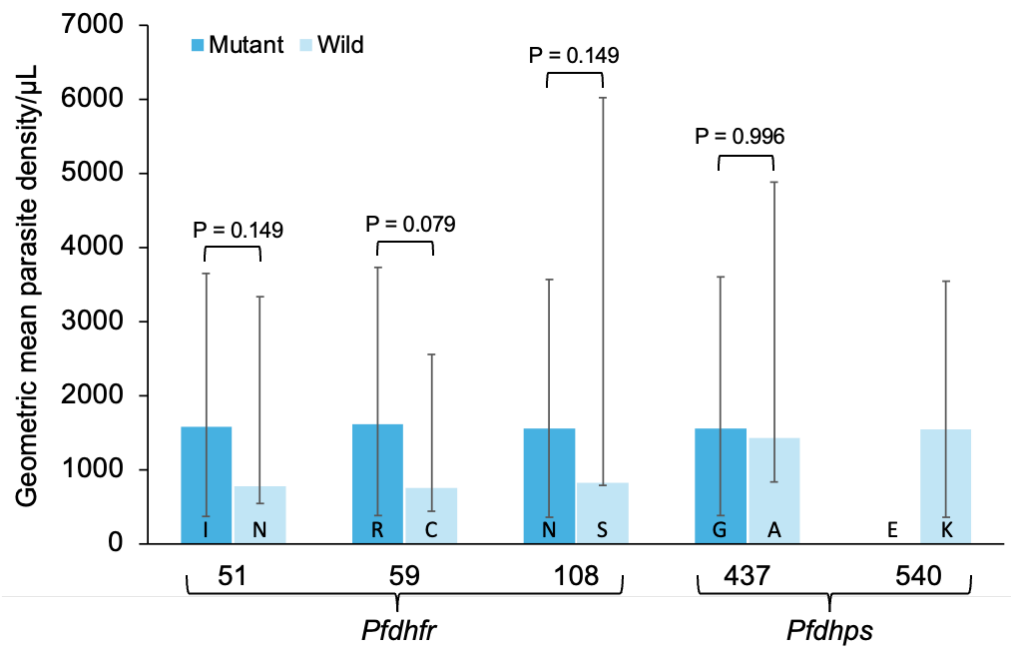
Comparison of geometric mean parasite density between wild and mutant alleles of *Pfdhfr* and *Pfdhps* genes. I= Isoleucine, N=Asparagine, R=Arginine, C= Cysteine, S=Serine, G=Glycine, A=Alanine, E= Glutamate, K=Lysine

There was no significant difference between the geometric mean parasitic density in samples carrying mutant alleles compared to those with the wild alleles either for *Pfdhfr* or *Pfdhps* (Figure 1).

## Discussion

This study reported high mutation frequencies in *Pfdhfr* 51I, 59R, and 108N as well as *Pfdhps* 437G irrespective of the period of the SMC campaign. Similar trends have been reported from Mali, Cameroon, Uganda and others regions of Burkina Faso [19–22]. These results may reflect the long history of SP usage. Indeed, before the large-scale implementation of ACTs in 2005, SP was used as first or second line treatment for uncomplicated malaria [23]. Subsequently, it was used for IPTp and more recently for SMC and IPTi. These changes have probably led to a progressive selection of these mutant alleles over time. These high prevalences could constitute a threat as few studies suggested that mutations in *Pfdhfr* have almost reached fixation [24] and may therefore have little odds to contribute further in the resistance phenotype [22] but may provide the genetic background for the expression of high-grade resistance. The presence of the *Pfdhps* 540E mutation, which is known to be associated with the resistance to SP was not detected in this study, consistent with previous studies [25,26]. This observation is not surprising because of the general finding that the K540E mutation is rare in West Africa [27]. The lack of *Pfdhps* 540E supports the excellent efficacy of AQSP in Burkina Faso [28]. However, similar studies carried out in Uganda and Mozambique reported a high prevalence of the *Pfdhps* 540E mutation before and after the SMC campaign [20,21,29]. This mutation is known as being very common in eastern and southern Africa [30].

No significant variation in the prevalence of the mutations was observed following the implementation of the SMC campaign. This trend has been reported in previous studies carried out in Burkina Faso, Uganda and Mozambique [19–21,29]. This suggests that these mutation frequencies are related to continuous use of the SP in interventions mentioned above over time.

An increase in the prevalence of triple mutant *Pfdhfr* was observed in the period before and after SMC, but this increase was not statistically significant. However, the *Pfdhfr* triple mutation was prevalent in more than 75% of samples both before and after SMC campaign. This study is in line with similar studies that have reported that about 75% of the *P. falciparum* parasite strains carrying the triple mutations in *Pfdhfr* [31]. Several studies showed that the *Pfdhfr* triple haplotype was highly prevalent in African isolates [32,33]. A number of factors such as the early emergence of the *Pfdhfr* triple mutant genotype in Africa [34], the pervasive drug pressure from the distribution of pyrimethamine for malaria prophylaxis in various parts of the world [35], and human migration patterns [36], may explain the greater distribution of this genotype throughout the continent [37]. However, it is well established that this haplotype does not correlate with in vivo SP treatment failure in West Africa [38,39], its detrimental effects may be largely compromised by an absence of the *Pfdhfr* I164L mutation. Like the triple mutation *Pfdhfr*, no significant increase in quadruple mutation *Pfdhfr-Pfdhps* was observed, though the prevalence before and after SMC campaign was high. Studies suggested that the saturation of the *Pfdhfr* triple mutants could further induce the *Pfdhps* mutants, and thus, the presence of quadruple mutants (CIRNI-SGKAA) was common [40]. The GE-IRN variant haplotype was not found in our study. Until now this haplotype has been very rare in West Africa due to the rarity of the *Pfdhps* 540 mutation [25]. In this study mutation carriage does not appear to have an influence on parasite density as this has been reported in other studies [23,40]. The limit of the present study is the number of samples genotyped which was limited to microscopy positive samples with the potential of missing rare haplotypes from subpatent parasitaemia. Additionally, position 431, 436, 581 and 613 of the *Pfdhps* gene are well known for their involvements in sulfadoxine resistance while these mutations were not assessed in the present study. Another limitation of this study is its short duration, allowing only the assessment of the impact of SMC over a single yearly campaign while the selective pressure could be very apparent with the repetition of the number of SMC campaigns over time.

## Conclusions

In Nanoro, the prevalence of *Pfdhfr* and *Pfdhps* mutant alleles was high, both before and after the SMC campaign. No significant variation in these prevalences was observed following the SMC campaign suggesting that this high mutation frequencies is related to the continuous and long-term use of SP in malaria prevention interventions. Continuous monitoring of resistance is necessary to preserve the effectiveness of antimalarial drugs and the impact of major drug-based prevention strategies.
